# The nexus of child undernutrition and household environmental conditions in Bangladesh: implications for public health and societal productivity

**DOI:** 10.1017/S1368980025100438

**Published:** 2025-06-03

**Authors:** John Patrick C. Toledo

**Affiliations:** Department of Theology and Religious Education, De La Salle University, 2401 Taft Avenue, Manila 1004, Philippines

**Keywords:** Undernutrition, Environmental condition, Child, Productivity

Dear Editor,

I’ve read with great interest the recently published study entitled Child Undernutrition and Its Association with Household Environmental Conditions in Bangladesh^(1)^. This study highlights child undernutrition as an alarming public health issue in Bangladesh, where a large percentage of children under five experience micronutrient deficiencies, stunting and wasting; these conditions are sometimes made worse by unfavourable home environmental factors, such as a lack of clean water, poor sanitation and crowded living arrangements.

The potential to inform successful interventions makes monitoring child undernutrition in connection to home factors a priority. Policymakers can create efficient initiatives to enhance living circumstances and nutrition by having a better understanding of how environmental factors contribute to undernutrition. In order to end the cycle of poverty and malnutrition, this is essential.

According to the WHO^(2)^, malnutrition increases healthcare costs, reduces productivity and slows economic growth, which can perpetuate a cycle of poverty and ill health. More than the economic aspect, undernutrition in children has serious repercussions. Long-term health issues brought on by child undernutrition include irreversible cognitive impairment and increased susceptibility to illness, which can hinder personal growth and lower general quality of life. These health issues also frequently feed a cycle of poverty and disadvantage that impacts not only the individual child but also their family and community for generations. Children who are malnourished are more prone to suffering from cognitive deficits, which can hamper their future productivity and educational success. In addition to the individual, the community and the country as a whole are also impacted by this cycle of poverty. Since a less productive workforce might result in slower economic growth, the long-term effects on the economy are likely to be substantial.

Furthermore, this work is relevant outside of Bangladesh. Poor household conditions are connected to high rates of child undernutrition in many other Asian nations, especially those with comparable socioeconomic issues. These problems are also present in nations like India, Pakistan and Myanmar; therefore, cross-border exchange of research and tactics is crucial to addressing this urgent public health issue.

In summary, child undernutrition in Bangladesh is an alarming public health issue associated with poor household environmental conditions, with detrimental societal consequences for the productivity of the young generation, relevant to other Asian countries facing similar challenges.

## Data Availability

The data that supports the findings of this study are available from the online references.
